# Strategies to mitigate muscle mass loss in individuals with spinal cord injury

**DOI:** 10.1080/10790268.2025.2503048

**Published:** 2025-06-06

**Authors:** Astrid M.H. Horstman, Janneke M. Stolwijk-Swüste, Luc J.C. van Loon, Sonja de Groot

**Affiliations:** 1 Human Performance Lab, Schulthess Clinic, Zurich, Switzerland; 2 Center of Excellence for Rehabilitation Medicine, University Medical Center Utrecht Brain Center, University Medical Center Utrecht and De Hoogstraat Rehabilitation, Utrecht, the Netherlands; 3 Department of Human Biology, NUTRIM Institute of Nutrition and Translational Research in Metabolism, Maastricht University Medical Centre+, Maastricht, the Netherlands; 4 Department of Human Movement Sciences, Faculty of Behavioural and Movement Sciences, Vrije Universiteit Amsterdam, Amsterdam Movement Sciences, the Netherlands

**Keywords:** Muscle protein metabolism, Anabolic resistance, Nutrition, Exercise mimetics, Pharmacological intervention

## Abstract

**Context:**

Muscle atrophy following spinal cord injury (SCI) is severe, often exceeding rates observed in disuse or critical illness. Neurogenic factors, such as the loss of neural input and disruption of neuromuscular signaling worsen the effects of physical inactivity. The severe muscle loss lowers muscle quality and impairs function, increasing the risk of chronic metabolic diseases. Understanding the mechanisms underlying SCI-induced muscle atrophy is crucial for developing targeted interventions.

**Objective:**

This review examines SCI-induced muscle atrophy mechanisms, addresses limitations of current interventions, and proposes therapeutic strategies integrating exercise mimetics, nutrition, and pharmacological agents to overcome anabolic resistance and improve muscle preservation.

**Methods:**

A PubMed search was conducted using terms including “spinal cord injury”, “disuse”, “muscle atrophy”, “anabolic resistance”, “muscle protein metabolism” and “interventions”. (Pre)clinical studies and reviews relevant to skeletal muscle physiology after SCI were included.

**Results:**

Anabolic resistance seems to be a key contributor to rapid SCI-induced muscle loss. Motor-complete SCI prevents voluntary contractions, limiting traditional exercise-based interventions. Neuromuscular electrical stimulation (NMES) can mitigate muscle atrophy during immobilization and critical illness especially when combined with protein supplementation. Additional nutritional interventions, including vitamin D, creatine, ursolic acid, polyunsaturated fatty acids (PUFAs), and pharmacological agents such as androgens, myostatin inhibitors, and β2-adrenoceptor agonists, may further attenuate muscle atrophy.

**Conclusion:**

A multidimensional approach integrating NMES, targeted nutrition, and pharmacological strategies may enhance muscle mass retention, metabolic health, and rehabilitation outcomes in individuals with SCI. Further research is needed to refine these interventions and establish clinical guidelines for muscle preservation in this population.

## Introduction

A spinal cord injury (SCI) leads to partial or complete loss of motor and sensory functions. The majority of affected individuals are male (±60%), have paraplegia (±60%), and experience a motor-incomplete SCI (±70%) and a non-traumatic cause of SCI (±65%) (1). An inactive lifestyle is often the result of an SCI. Muscle atrophy is severe, especially with a sustained lack of voluntary neural muscle activation, as seen in individuals with motor-incomplete SCI. Leg muscle cross-sectional area (CSA) declines by 40–45% within six weeks following injury. Continued muscle loss over time leads to approximately 55% loss of leg lean mass in individuals with a motor-incomplete SCI, and about 60% in those with a motor-complete SCI (2,3). This loss of muscle mass is accompanied by a decline in muscle quality, compromising functional and structural properties of muscle that determine its strength, endurance, and metabolic efficiency. Specifically, it includes increased intramuscular fat infiltration, a shift toward fast-twitch, fast-fatigable muscle fibers (which are less efficient and more prone to fatigue), and reduced mitochondrial density and oxidative capacity(2,4–7).

Beyond its primary locomotor function, skeletal muscle performs many vital metabolic functions. It contributes to resting metabolic rate (8–10). Extensive loss of muscle mass and a concomitant increase in body fat mass following SCI (3,11) negatively impact resting metabolic rate and peripheral glucose utilization (12,13). These changes increase risk factors for developing cardiovascular disease, such as chronic low-grade inflammation and metabolic syndrome (14). This contributes to a higher incidence of cardiovascular disease, the primary cause of mortality among individuals with SCI. The co-existence of excess adiposity and low muscle mass/function, called sarcopenic obesity, has been associated with poorer health outcomes and lower quality of life than sarcopenia and obesity alone (15–17). It is shown to be prevalent in approximately 40–65% of the SCI population (18,19). Moreover, muscle disuse atrophy has been associated with the development of deep pressure ulcers in SCI (20–23), resulting in high health care costs and decreased quality of life. Altogether, this underscores the urgent need to find ways to counteract muscle mass loss – in individuals with a motor-complete SCI, who may not be able to use part of that muscle mass functionally.

This review addresses the mechanisms of muscle atrophy post-SCI. It also evaluates evidence-based interventions that may enhance muscle protein synthesis, reduce muscle protein breakdown, and mitigate or partially reverse the loss of muscle mass and function. We address (combined) neuromuscular electrical stimulation, nutritional supplementation, and pharmacological interventions and their limitations. These may help mitigate muscle mass loss in paralyzed muscle, potentially improving the general (metabolic) health and well-being of individuals with SCI.

## Muscle mass loss in SCI

**Figure 1:**
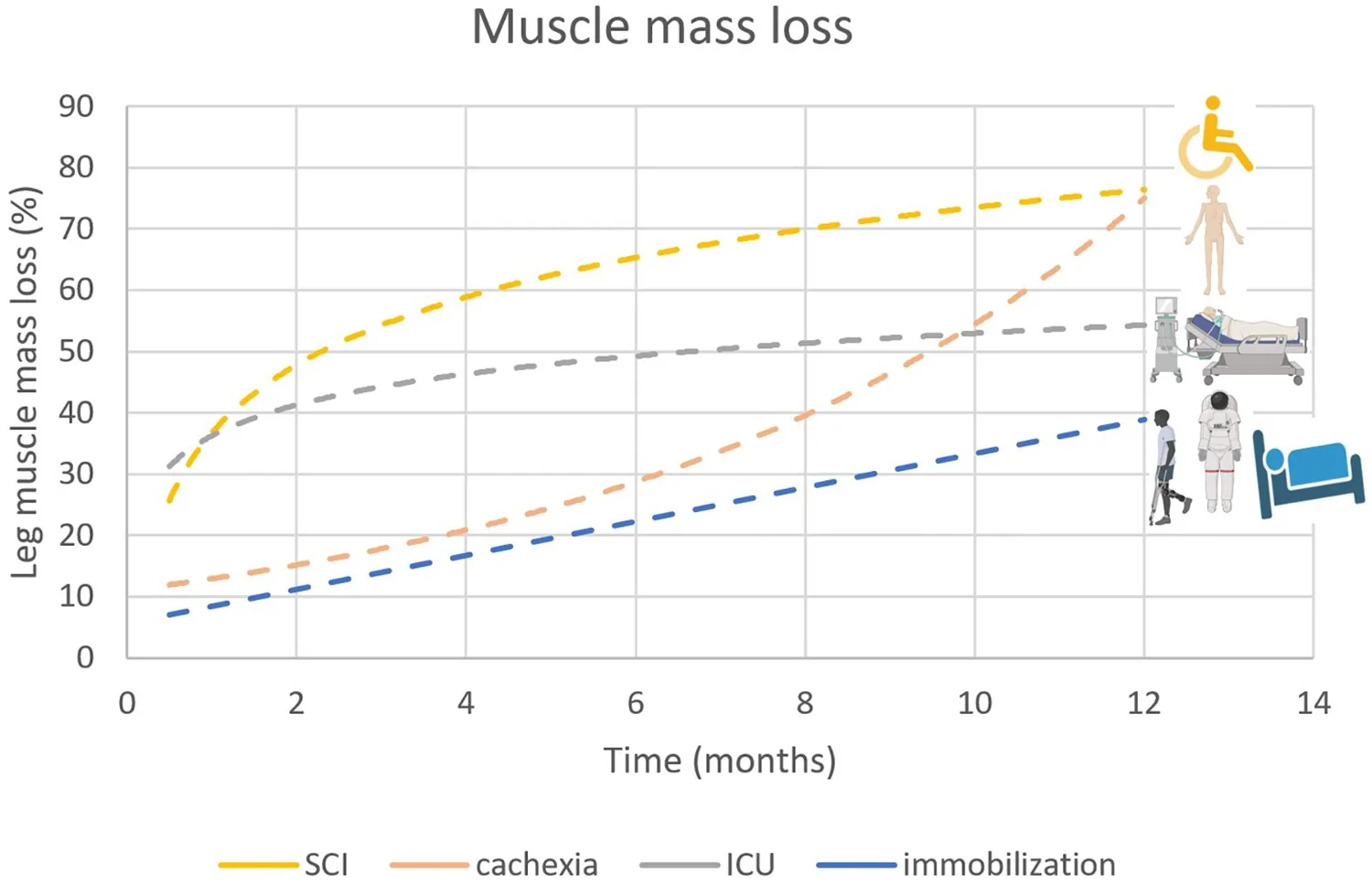
Rate of muscle loss over time after spinal cord injury (SCI), during cachexia, stay at the intensive care unit (ICU) and immobilization. The rate of muscle loss in the group with SCI is significantly higher compared to the other three groups.

In individuals with motor-complete SCI, the average lower leg CSA has been reported to be 45–80% of that in age – and weight-matched able-bodied controls at 24 weeks after injury (2). One year post-injury, muscle mass seems to have reached a plateau (24). This pattern seems most comparable with people at the ICU, who also lose most muscle mass in the first few weeks of hospitalization, reaching a plateau at around 50% muscle lost at 4–6 weeks (25). In models of disuse atrophy such as bed rest and limb immobilization, we see a more modest linear loss of muscle mass (26,27). Following 5 and 14 days of limb immobilization, a respective 4% and 8% loss of muscle mass has been reported (28,29) . After 4–8 weeks of bed rest, a 10–16% (26,30,31) loss of muscle mass has been observed. During even longer periods of disuse, such as 17 weeks of bed rest (27,32) and 16 – to 28-week space flights (shuttle/Mir), a decrease of 6–20% in muscle mass (33) is seen. Muscle mass loss then levels off (27,34–45) reaching ∼40% (46). In cachexia, muscle mass loss accelerates, peaking in the last three months (47), reaching up to 75% (48). We have visualized the amount of muscle loss over time in the four situations – immobilization, cachexia, ICU and SCI – in Fig. 1. The decline in muscle mass following SCI is typically much greater than that observed after limb immobilization or bedrest of able-bodied individuals (42,49). The greater muscle mass loss after SCI is contributed to several factors. Immediately after injury, individuals often enter a phase of spinal shock (50,51). This is a sudden and temporary loss or impairment of spinal cord function. It occurs below the level of injury after an acute SCI. This condition includes an abrupt loss of synaptic input to motor neurons below the level of injury (52). This neural silencing accelerates the decline in muscle activity and subsequent atrophy. Unlike in immobilization, where muscle innervation is maintained, motor-complete SCI results in temporary denervation of affected muscle groups, significantly accelerating muscle loss. In the subacute or chronic phase of motor-complete and incomplete SCI, spasticity may occur, resulting in involuntary muscle contractions. Although this introduces some level of muscle activation, it is generally insufficient to maintain muscle mass and may even contribute to fatigue, pain and contractures. Long-term denervation also induces structural and cellular changes, including reduced satellite cell activation, loss of neuromuscular junction integrity, and increased fibrotic tissue deposition, as well as hormone level fluctuations and inflammation, all of which further impair muscle regenerative capacity after SCI (53).

The timelines of muscle loss in Fig. 1 are merely estimated based on the existing literature. Other reports indicate less muscle mass loss in SCI, which may be attributed to *e.g.* the duration of injury, the level and completeness of lesion (54,55) and occurrence of spasticity. In the ICU, large variations exist in reported muscle mass loss. Factors such as height, age (25), and the presence or absence of multi-organ failure strongly impact the rate of muscle mass loss in critically ill individuals (56). In addition, people with greater muscle mass at baseline generally lose significantly more muscle thickness (57). Patients with low muscle mass and those admitted to the ICU with a body mass index of 35 kg/m^2^ have poorer clinical outcomes (58–60). In patients with cancer, muscle mass loss strongly depends on treatment and nutritional status (61). Furthermore, sex has also been shown to play a role in the time course of muscle loss, with women subjected to bed rest losing significantly more muscle mass at a more rapid pace than men (34–36,45). Whether all these factors also affect the level and timing of muscle mass loss in SCI remains unclear.

### Muscle protein turnover

Muscle fiber CSA has been suggested to decline by approximately 60% between 1 and 17 months following injury (62), after which muscle mass seems to stabilize (63–66). Skeletal muscle mass loss is underpinned by a chronic imbalance between muscle protein synthesis and breakdown rates. Muscle atrophy during short-term disuse, *i.e.* the initial rapid phase, is primarily attributed to a sharp increase in muscle protein breakdown rates and a reduction in muscle protein synthesis. In line, in SCI the expression of protein degradation markers, MuRF1 and MAFbx, have been reported to be significantly increased as early as two days following spinal cord injury (67).

Muscle atrophy during more prolonged (>10 days) disuse is caused primarily by declines in basal and postprandial muscle protein synthesis rates, without significant changes in muscle protein breakdown. Following an SCI, a significant loss of skeletal muscle, accompanied by an increase in fat mass, is reported to occur within a short time after the event (3,11), especially when compared to pure disuse atrophy (Fig. 1). To date, no *in vivo* human data on SCI-related muscle protein synthesis and muscle protein breakdown are reported. However, studies in denervated rats show either increased (68) or decreased muscle protein synthesis (69,70) and muscle protein breakdown rates may remain elevated in long-term paraplegia (71). Data on the potential changes in muscle protein synthesis and breakdown rates during the early and latter stages following SCI are lacking in humans. It is possible that the changes in muscle protein turnover during the early stage of SCI gradually diminish, leading to a new steady-state condition of reduced muscle protein turnover, in which further muscle atrophy is halted.

In disuse atrophy, we see a decrease of approximately 30–40% in basal protein synthetic rates (28). In an earlier publication, we showed that alterations in basal or postprandial fractional muscle protein synthesis and breakdown rates as small as 1%–2% can already induce a decline in body weight of more than 5% within six months (72). As shown in Fig. 1,muscle loss in patients with cachexia has been reported to reach up to 75%, similar to the amount of muscle atrophy seen in SCI. In people with SCI, no data on *in vivo* muscle protein synthesis and breakdown rates are available. However, based on a muscle mass loss of 45–80% during the first year after injury (2) and using the same reasoning as in Horstman *et al.* (72), we can calculate the theoretical changes in basal muscle protein synthesis or muscle protein breakdown rates required to induce such a decline in muscle mass after SCI. This would mean a decrease in rate of basal muscle protein synthesis or increase of basal muscle protein breakdown rates as small as 0.5–0.9% (365 days, 8760 h). Besides changes in basal muscle protein synthesis and muscle protein breakdown rates, alterations in postprandial muscle protein synthesis rates may play an even bigger role in the severe muscle atrophy seen after SCI. The loss of muscle mass with aging (73), obesity, and cancer (74) have been largely attributed to a blunted muscle protein synthetic response to protein feeding. In patients in the ICU, the muscle loss seems to be attributed mainly to a decline in postprandial muscle protein synthesis, as basal muscle protein synthesis rates do not seem to be impaired (75). Therefore, muscle losses in disuse, aging, cancer, and ICU have all been attributed, at least for a large part, to a blunted muscle protein synthetic response to dietary protein feeding, now coined anabolic resistance. The etiology behind anabolic resistance remains to be established, as it could include compromised protein digestion, amino acid absorption, postprandial perfusion, amino acid uptake, muscle anabolic signaling, and tissue protein synthesis. So far, studies in the ICU suggest that protein digestion and amino acid absorption are unlikely a key factor in the compromised ability to respond to protein feeding. Therefore, the mechanisms responsible for anabolic resistance are likely intrinsic to skeletal muscle tissue metabolism.

### Fast vs slow twitch muscle

It is well-known that paralyzed muscle gradually changes to a fast-fatigable phenotype (predominantly type IIX fibers) (65,76–79) with less oxidative enzymes (65), decreased skeletal muscle mitochondrial capacity (65,80,81), increased fatigue (77,82–85), and muscle atrophy (2,4). The change from oxidative slow-twitch to glycolytic fast-twitch fiber types is a hallmark of tissue atrophy (86). From 1 to 20 months following SCI, muscle fibers gradually shift from expressing predominantly slow myosin heavy chain (MHC) isoforms to a higher proportion that co-expresses both fast and slow MHC (62–64,87,88). By ∼70 months post-injury, its nadir is reached where nearly all muscle fibers predominantly express the fast MHC isoform, indicating a shift toward faster, more fatigable muscle properties (89). Although fiber type composition *per se* may not be a major factor that influences atrophy after SCI in humans (2), the shift in fiber type (88–90) combined with the overall muscle loss, impairs the metabolic flexibility of the muscle of individuals with SCI. This means that the muscle fibers lose some of their capacity to rapidly switch between glucose and fatty acid utilization as fuel for ATP generation. This change in muscle energy metabolism can lead to a decrease in overall metabolic health and increase the risks of developing chronic metabolic disorders such as (sarcopenic) obesity, dyslipidemia, insulin resistance, type 2 diabetes, and cardiovascular disease (19,91,92). This may also imply that interventions promoting an increase in muscle mass and mitochondrial capacity could improve overall metabolic flexibility and (muscle) health of individuals with SCI.

## Passive mobilization and exercise mimetics

Skeletal muscle mass after SCI can decline to 50% of able-bodied individuals (4) and plays a crucial role in increasing the risk of premature all-cause mortality (93). Resistance exercise training is well-known to stimulate gains in muscle mass and strength, to reduce systemic inflammation, and enhance longevity and quality of life (94–96). However, such training may not be feasible for patients with neuromuscular injuries or diseases, such as SCI. Individuals with a motor-complete SCI are unable to voluntarily activate a significant portion of their total skeletal muscle mass. In this paragraph, we will describe the use of passive mobilization and exercise mimetics that have been used to overcome muscle weakness and (partial) denervation, aiming to improve muscle mass and the associated physical health benefits in individuals with SCI (97–99).

### Passive mobilization

Seventy years ago, animal studies showed that passive stretch either promotes growth or reduces atrophy in innervated (100,101) as well as in denervated (102,103) muscle. This could be, at least partially, explained by an increase in muscle protein synthesis rates, since the fractional rate of synthesis was significantly elevated (40%) in the stretched muscle (104). Stretching was adopted as a rehabilitative strategy largely due to studies showing it to be effective at maintaining range-of-motion (ROM) and preventing joint contractures for immobilized, non-injured animals (105,106). Nowadays, stretching and ROM exercises are routinely initiated in individuals with SCI, once patients’ vital signs are stable with the goal of preventing or reducing secondary complications such as spasticity and contractures (107,108). Moreover, poor hamstring muscle extensibility is a common problem in people with SCI (109,110). Therefore, physiotherapy is often directed at increasing extensibility in these muscles (108). A systematic review on human studies also investigated the effect of passive stretching on ROM, showing that the effect of stretching on ROM is limited or short-lasting (111,112). In addition, multiple sessions of passive leg cycling in persons with SCI lead to increases in myofibrillar protein content (113) and genetic expression of MHC filament proteins (MHC types IIa and IIx), and a concomitant decrease in genetic expression of markers of protein degradation (ubiquitin) (114,115). These results suggest that passive cycling has the potential to attenuate muscle atrophy. However, further studies are needed to determine whether passive cycling can protect muscle mass and to assess the effect of passive stretching in general on (counteracting) changes in muscle protein synthesis and muscle protein breakdown rates and, subsequent muscle mass loss in individuals with SCI.

### Neuromuscular electrical stimulation (NMES) and functional electrical stimulation (FES)

Surface neuromuscular electrical stimulation (NMES) delivers repeated electrical pulses to produce contraction of innervated muscles after depolarizing local axonal branches. A single session of high-frequency, high-intensity NMES increases (fasting) muscle protein synthesis rates (116) and has been effectively applied to prevent muscle atrophy in various clinical conditions (117–125), including non-sedated ICU patients (126), fully sedated comatose ICU patients (127) and healthy individuals subjected to limb immobilization (125). Specifically in SCI, NMES has been shown to increase muscle fiber size and type (76,128–130), as well as whole muscle CSA (96,131–139) and tetanic muscle strength (135). A systematic review showed that NMES-based resistance training provides the most robust and consistent evidence for increasing skeletal muscle mass in paralyzed limbs of adults with motor-complete SCI, compared to other NMES applications, functional electrical stimulation (FES, *i.e.* the use of electrical current to directly enable functional or coordinated movements), and pharmacological treatments (140). A meta-analysis showed that NMES and/or FES increase muscle mass in SCI, with muscle CSA increases ranging from 6% to 75% and an average increase of 26% (134). Higher training volumes, such as four sets of electrically-evoked resistance exercise or 5 sets of 10 isometric repetitions, were most effective in inducing muscle hypertrophy (134). NMES-induced increases in basal muscle protein synthesis rates are supported by upregulation of muscle hypertrophy markers Akt and mammalian target of rapamycin (mTOR) after 12–16 weeks of training in persons with chronic SCI (141). Moreover, NMES may also attenuate muscle protein breakdown, as indicated by downregulation of protein degradation markers myostatin and MurF-1(141). The trained paralyzed muscle also shows a shift from fast-twitch to oxidative glycolytic fibers (142).

In addition to improving muscle mass, NMES (preferably 2–3 times weekly) enhances metabolic profiles (97,131,138,143), by enhancing glucose metabolism and insulin sensitivity (142) and reducing intramuscular fat (136,137). Aerobic or resistance exercise training with NMES has shown promising results for metabolic improvements (97,138,139,144) though the optimal exercise prescription remains unknown. Neither has it been investigated whether NMES increases muscle protein synthesis in individuals with a motor-complete SCI. Future studies should also examine the effects of NMES on prevention of secondary outcomes such as spasticity control, contracture prevention, and ulcer prevention (145,146), quality of life, and long-term outcomes such as morbidity and mortality. Finally, when applying NMES to individuals with SCI, healthcare providers must be vigilant about specific risks, including autonomic dysreflexia (a potentially life-threatening condition), musculoskeletal injuries (*e.g.* fractures and dislocations), post-exercise hypotension, and thermal dysregulation, which are due to the autonomic dysfunction common in patients with SCI (147). Combining passive mobilization with NMES or FES could provide a synergistic effect, as animal studies have shown that such combinations may prevent both muscle atrophy and the accumulation of connective tissue, (148,149), offering a promising avenue for improving functional outcomes in SCI rehabilitation.

## Nutrition

Though it is well established that nutrition plays a crucial role in maintaining muscle mass (150,151), less attention has been given to developing effective nutritional strategies to attenuate or even prevent muscle loss during periods of muscle disuse, such as after SCI. Total daily energy expenditure after SCI is reduced by 12% to 54%, depending on the level and completeness of injury, fat-free lean mass, and activity level. In addition, the population with SCI faces challenges in participating in physical activity. Approximately 50% of people with SCI experience malnutrition (152,153), defined as “deficiencies, excesses or imbalances in a person’s intake of energy and/or nutrients” (154). Malnutrition includes both undernutrition as well as overweight, obesity and diet-related noncommunicable diseases (such as heart disease, stroke, diabetes, and cancer). If untreated, malnutrition can lead to longer hospital stays, increased in-hospital mortality, pressure ulcers, suboptimal functional recovery, obesity and other cardiometabolic issues (155). Therefore, an appropriate diet is of paramount importance for individuals with SCI (156,157). And although the risk of obesity is higher in the chronic phase compared with undernutrition, it is important to address both ends of the nutritional spectrum (158).

### Energy balance and macronutrient composition of the diet

During the acute phase of SCI (0–4 weeks), metabolic alterations such as reduced basal energy expenditure, increased nitrogen excretion, and weight loss are common (159–161), often resulting in negative nitrogen balance despite nutritional support. In the post-acute phase, up to 47% of patients are at risk of undernutrition, while overweight and obesity affect up to 80% (162). In the chronic phase post-discharge, metabolic syndrome is highly prevalent, especially in individuals with tetraplegia (163). Significant muscle mass loss in chronic SCI leads to a reduction in basal metabolic rate (BMR), which forms around 65–70% of daily energy expenditure. The location and completeness of the injury determines the extent of its effect on the BMR, such that individuals with motor-complete tetraplegia have a lower metabolic rate than those with paraplegia (164–166). This lower BMR (167–169), combined with a decline in physical activity (163,170,171), often creates an energy imbalance that contributes to fat mass gain, reduced lean mass, and a decline in overall metabolic health (11,172–176). Studies comparing metabolic rate and total caloric intake demonstrate an excess of 300–500 kcal/day in persons with chronic SCI (170,177,178). According to Farkas *et al.*, carbohydrates, proteins and fats account for 44% to 53% (177,179–181), 17% to 19% (97,170,182), and 34% to 40% (97,182–185), respectively, of the total energy intake in the population with SCI (186). Many individuals with SCI consume diets high in fats and carbohydrates (187), which can elevate insulin levels, promote fat accumulation, and increase inflammation, hypertension, and the risk of obesity and its associated complications (168,188). Adjustments to the diet – favoring high-protein, low-carb intake – might improve body composition and metabolic health, as protein is more satiating, even with similar or lower energy intake (189). In addition, dietary protein is well known to stimulate muscle protein synthesis rates (190–192), preserve fat-free mass (193) and improve insulin sensitization (194). Tailoring energy, protein, and micronutrient intake to the specific needs of individuals with SCI could be crucial for preserving muscle mass, optimizing metabolic health, and preventing secondary complications, such as pressure ulcers (195–197).

### Supplementation with protein

Not only declines in fasting muscle protein synthesis rates (28,198–200), but also a reduced responsiveness to the anabolic properties of amino acids/food intake, known as anabolic resistance (200–202), contributes to disuse atrophy. On the other hand, dietary protein intake stimulates muscle protein synthesis by supplying amino acids to the muscle (190,191). The postprandial muscle protein synthetic response is impacted by several factors, including dietary protein digestion, amino acid absorption (203,204), splanchnic amino acid extraction (205,206), plasma amino acid availability, vasodilation, the delivery of amino acids to the muscle (207,208), uptake of amino acids by the muscle (209,210), and intramuscular signaling that activates the anabolic response of muscle protein synthesis (211,212). As a result, the amount (213–216), source (217–220), and type (203,221,222) of ingested protein uniquely influence plasma amino acid levels and, consequently, affect both the extent and duration of the postprandial increase in muscle protein synthesis rates. Also the timing of protein intake during the day (223) or relative to exercise (224) modulates muscle protein synthesis rates. Whether dietary protein digestion, amino acid absorption, and amino acid appearance in blood is affected by the SCI, and whether the level of anabolic resistance is greater after SCI compared to disuse alone, remains to be assessed. To the best of our knowledge, muscle protein synthesis rates and the effect of nutrition on them have never been measured in individuals with SCI. What is well known, however, is that dietary protein is essential to maintain skeletal muscle mass in a setting of a sedentary lifestyle with low physical demands, such as after SCI (225). An individual’s protein requirement is defined as the lowest amount of habitual dietary protein intake that will balance body nitrogen loss in individuals maintaining energy balance (225). Research on protein requirements for individuals with SCI has focused primarily on the acute injury phase (165,226,227), with limited data on the needs during chronic SCI. The Academy of Nutrition and Dietetics suggests a daily protein intake of 0.8–1.0 g/kg body weight (BW) for people with chronic SCI in the absence of pressure ulcers or infection. Farkas *et al.* indicated that only 16% of individuals with chronic SCI meet these guidelines, with intake decreasing as BW increases, leading to underconsumption in 85% of individuals above 72.4 kg (228). Persistent protein underconsumption can accelerate fat-free mass (FFM) loss and elevate fat mass, especially as basal metabolic rate (BMR) decreases. Conversely, high-protein diets can protect FFM during energy restriction, potentially mitigating BMR decline (229,230). Such diets may also reduce the risk of obesity in people with SCI (231), due to the satiating effect of protein (189,232).

Also many individuals with SCI fall short of recommended intakes for certain essential amino acids (177,233,234). These deficiencies are significant, as essential amino acids are those that the human body cannot synthesize in sufficient quantities and must therefore be obtained through dietary intake. Essential amino acids, and leucine in particular, have the capacity to activate the mRNA translational machinery via mTOR (235,236). Leucine plays a critical role in intracellular signaling and directly influences skeletal muscle protein metabolism. It serves as a key regulator of postprandial muscle protein synthesis and must be available in adequate concentrations, alongside other essential amino acids, to maximize the muscle protein synthetic response following nutrient intake (237). The essential amino acids are critical for tissue repair and protein synthesis, processes crucial for maintaining muscle mass and managing pressure injuries in people with SCI. Pressure injuries after SCI precipitously deplete the limited protein stores as the body attempts to heal the wound, generating a rapid transition to malnutrition (238). This occurs in the presence of already markedly diminished protein reserves (*i.e.* skeletal muscle mass). Therefore, investigating the optimal amount, type, and timing of dietary protein or amino acid intake to increase muscle protein synthesis rates and, consequently, muscle mass is of utmost importance, as well as assessing the specific amino acid needs *e.g.* during urinary tract and pulmonary infections and in repairing ulcers and other pressure injuries. In addition, changes in the gastro-intestinal tract (GI) tract, such as neurogenic bowel syndrome, slower bowel movement and changes in microbiome after SCI, most likely cause changes in digestion and absorption, which may also lead to specific protein but also other macro – and micronutrient needs.

Future studies should investigate questions such as: what is the turnover rate of immobilized or denervated muscle tissue in individuals with SCI and how does this affect dietary protein requirements? What has changed in the protein digestion and amino acid absorption kinetics? How much and what type of protein would be needed to optimally stimulate muscle protein synthesis? Would free amino acids improve exogenous amino acid availability more compared with intact protein? Would there be a synergistic effect on muscle protein synthesis if dietary protein intake is combined with NMES?

In able-bodied persons as well as in different patient populations, it has been shown that the combination of exercise (mimetics) and dietary protein intake enhances the increase in muscle protein synthesis compared to exercise or nutrition alone. A single bout of exercise transiently stimulates muscle protein synthesis rates and, to a lesser extent, muscle protein breakdown rates (239,240). However, without nutritional support, the net muscle protein balance remains negative (240). Protein ingestion following exercise enhances muscle protein synthesis and suppresses muscle protein breakdown, leading to a net positive protein balance right after exercise (241). Consequently, post-exercise protein consumption is a widely adopted strategy to enhance muscle protein synthesis and, in the longer term, to improve muscle mass and strength. It has recently been shown that the positive effect of resistance training using neuromuscular electrical stimulation (NMES-RT) on thigh lean mass was potentiated by protein supplementation in individuals with chronic SCI (242).

### Supplementation with other nutritional compounds

Besides proteins, the macronutrients fat and carbohydrates are also important in the diet, both in general and specifically in SCI, as malnutrition is an independent risk factor for sarcopenia and doubles the risk of pressure ulcers (195). Deficiencies in vitamins A, B5, B7, C, D, and E and minerals calcium, magnesium, and potassium are common in individuals with chronic SCI (179,180,183,185,233).

#### Vitamin D

Vitamin D is known to affect muscle function (243,244), and suboptimal vitamin D status is prevalent in up to 93% of individuals with SCI, both directly after SCI onset (245,246) as well as in chronic SCI (247), which is significantly higher compared to up to 40% in the general population (248,249). This increased prevalence can be explained by the adverse impact of SCI on physiological function, including altered metabolism and gastrointestinal functioning, as well as lifestyle behaviors, such as reduced physical exercise, outdoor activity, and sun exposure (248,250). The evidence regarding the effects of vitamin D supplementation in individuals with SCI remains inconclusive. One study found that vitamin D supplementation successfully achieved sufficient vitamin D levels in 91% of elite athletes with SCI, with 62% demonstrating improved muscle strength (251). However, another study reported no significant increase in serum vitamin D levels among patients with SCI during inpatient rehabilitation following supplementation. These contrasting findings highlight the need for further research to clarify the role of vitamin D in this population (252).

#### Creatine

Creatine supplementation in the aging population has been shown to increase the availability of creatine and phosphocreatine in muscle, which may promote the expression of growth factors like insulin growth factor (IGF) 1 and the phosphorylation of key signaling proteins (253). Creatine supplementation can be used to increase muscle mass and enhance exercise capacity in adolescents, adults, and vulnerable older people and may be beneficial in muscle disorders like dystrophies, inflammatory myopathies, and cytopathies (254). The effect of creatine supplementation on immobilized muscle has been shown to either worsen (255–257) or fail to preserve (258) losses in muscle mass and strength during immobilization in healthy volunteers. However, creatine supplementation can have a synergistic effect with resistance training, leading to greater skeletal muscle changes than training alone (259–261). During rehabilitation of disuse atrophy, oral creatine supplementation in combination with strength training was shown to stimulate muscle hypertrophy and maximal knee extension power (257). Therefore, the combination of creatine with exercise (mimetics) may still be beneficial in preventing further muscle atrophy after SCI.

The review by Stojic *et al.* (262) analyzed eight studies on dietary supplements for musculoskeletal and physical health in SCI. Creatine or vitamin D supplementation, combined with resistance training, improved arm muscle area and strength (263), while short-term creatine supplementation improved peak power output during arm ergometry tests in persons with tetraplegia (264). However, creatine supplementation did not improve isometric strength of the wrist extensors nor hand function (Grasp and Release Test) in those with tetraplegia and mild wrist weakness (265).

#### Ursolic acid (UA)

Kunkel *et al.* (266) identified ursolic acid (UA), a natural plant metabolite with previously unrecognized anabolic properties, as a compound that reverses changes in human skeletal muscle mRNAs after SCI (266) and, subsequently, as an inhibitor of denervation-induced muscle atrophy (267). In addition, UA also reduced adiposity (267). In another, preclinical study, UA supplementation was shown to repress the expression of atrophy-related genes FOXO1 protein and MAFbx (protein breakdown) during the first week after SCI and prevented the SCI-related suppression of key proteins in the PI3 K/Akt/mTOR signaling (protein synthesis) pathway for several weeks thereafter (268). However, UA also promoted some locomotor recovery following SCI, which may have impacted these outcomes. UA supplementation may be useful as a monotherapy for illness – and age-related muscle atrophy or in combination with other strategies that have been considered, such as myostatin inhibitors (269). While promising, it remains unclear whether UA preserves muscle (fiber) CSA or mass in the paralyzed limbs of individuals with SCI.

#### Polyunsaturated fatty acid (PUFAs)

Omega-3 polyunsaturated fatty acids (Ω3-PUFAs) such as eicosapentaenoic acid (EPA) and docosahexaenoic acid (DHA) have been shown to stimulate muscle protein synthesis via increased activation of the mTOR-p70s6k signaling pathway and reduction of insulin resistance and anti-inflammatory effects that support anabolic processes (270,271). Animal studies have shown that fish oil, rich in PUFAs, enhances anabolic signaling and increases muscle protein mass (272,273). In rats with SCI, n-3 PUFAs were also shown to be able to inhibit mTORC1 signaling and enhance autophagy, promoting functional recovery (274). A systematic review in rats showed that DHA can promote motor functional recovery after SCI in rats (275). However, only a small number of studies were included of mainly low quality. In addition, there may be other potential neurological benefits of Ω3-FAs (276,277).

In humans, PUFA has been reported to increase both basal and postprandial muscle protein synthesis rates (270,278,279) and may be applied to mitigate anabolic resistance. In support, more prolonged PUFA supplementation has been reported to augment the benefits of resistance exercise training to increase muscle mass in older adults (270,279–282). Higher dietary intake or elevated plasma and red blood cell levels of Ω−3 fatty acids (FAs) were shown to be positively associated with improved muscle mass, strength, and function (271,283). Furthermore, Ω−3 PUFA supplementation has shown potential in mitigating muscle atrophy associated with aging, advanced cancer, and chronic diseases (284–286). Due to their anti-inflammatory properties, PUFAs may also play a protective role against inflammation-driven muscle wasting, such as cachexia and post-intensive care syndrome. A daily dose of 3 g of EPA and DHA was shown to increase muscle mass and function (287) as well as resting and exercise metabolic rate (288) in older adults. Although clinical trials examining PUFA supplementation in individuals with SCI are lacking, the potential neurological benefits and its effect on muscle mass and function and metabolism are promising.

To conclude, after SCI a lower energy and nutrient “setpoint” is reached compared to pre-injury levels (156,167–169). However, this reduced setpoint is rarely accompanied by a proportional decrease in energy intake (170). Excessive calorie consumption relative to energy needs, along with poor dietary habits, contribute to inadequate nutrition and chronic health issues among individuals with SCI (163,171). Consequently, ensuring the appropriate intake of macronutrients and micronutrients to meet altered needs in combination with changes in the GI tract after SCI remains a challenge, despite existing dietary recommendations. Moreover, personalized nutritional strategies should account for individual factors like age, sex, body composition, and activity level. For muscle health, special attention should be given to dietary protein as well as vitamin D, creatine intake, UA and PUFAs. Particularly, the combination of high-quality proteins, leucine, vitamin D, and Ω−3 PUFAs has been identified as a potential strategy for preventing muscle atrophy (289) and may be efficient in SCI as well.

## Pharmacological

Although no drugs are currently approved to combat muscle atrophy following SCI, and no pharmacological approach has been proven effective to reduce muscle loss in the paralyzed limbs of individuals with SCI, some (preclinical) studies on anabolic agents and pharmaceuticals suggest that these may help attenuate severe muscle loss in the paralyzed limbs. Though it should be noted that many of the anabolic agents also come with potential harmful side-effects.

### Androgens

Skeletal muscle disuse is accompanied by reduced basal, post-absorptive muscle protein synthesis rates, increased muscle protein breakdown rates, and anabolic resistance to nutrient intake (290,291). The anabolic-androgenic steroid nandrolone has been shown to increase muscle protein synthesis rates, decrease muscle protein breakdown rates, and attenuate denervation atrophy (292,293). In spinal cord-transected rats, it has been shown that nandrolone administration normalizes determinants of muscle mass and fiber type (294). In humans, long-term administration of testosterone or nandrolone (decanoate) (200–2400 mg over 1–24 months) has been associated with increases in fat-free mass, muscle CSA, and/or strength in various pathological conditions (295–301). Shortly after SCI, most men experience hypogonadism (302), with 40% of men with chronic SCI exhibiting low testosterone levels (303). Men with SCI and lower testosterone levels have been shown to have 29% more body fat and 72% more visceral adiposity, making them more prone to metabolic dysfunction compared to those with normal levels (304). A moderate dose of testosterone supplementation (5–10 mg/day for one year) was shown to increase whole body and lower extremity lean mass in hypogonadal men with chronic motor-complete SCI (305). Also three other studies found a positive effect of androgen treatment on skeletal muscle mass in individuals with SCI (136,306,307). In contrast, lower doses of 2–6 mg/day for four months did not influence whole body lean mass, lower extremity muscle CSA or muscle fiber CSA (308) in eugonadal and hypogonadal men with chronic motor-complete SCI. This seems in line with Horstman *et al.*, who found that a 200 mg dose of nandrolone decanoate did not prevent muscle loss when administered before seven days of disuse in able-bodied subjects (309). It seems that some type of exercise is needed for androgens to have an anabolic effect. Indeed, four months of testosterone replacement therapy combined with resistance-type exercise training in individuals with SCI, resulted in fiber hypertrophy and beneficial changes in markers of skeletal muscle function and health after SCI (308). However, it is unclear from this four-months study whether the testosterone administration had an additional effect compared to resistance type exercise training only. So, exercise and testosterone supplementation seem to have a synergistic effect and could be a promising approach to prevent or attenuate disuse atrophy and to improve muscle quality and metabolic dysfunction in individuals with SCI (308,310,311). RCTs are currently underway to investigate the effects of testosterone and long pulse width stimulation, as well as the combined effects of home-based functional electrical stimulation during leg cycle and arm ergometry with testosterone, on muscle mass and strength, metabolism, and well-being in both men and women with SCI (312,313). A case study showed that combining a standard daily diet plan (45% carbohydrate, 30% fat, and 25% protein – with a reduction of caloric intake by 45% and fat percentage by 6.5%) alongside a daily transdermal testosterone dose of 4 mg/d for 16 weeks, led to an 8% reduction in body weight and a 29% decrease in body fat, as well as increases in whole-body lean mass and absolute thigh muscle CSA by 7% and 9%, respectively. Though, caution is required regarding the potential health risks of high-dose testosterone supplementation, such as polycythemia (erythrocytosis), reduced high density lipoproteins (HDL) cholesterol, increased level of low-density lipoproteins (LDLs), elevation in liver enzyme, increased incidence of prostate or lower urinary tract-related events, increased irritability or mood changes, injection-site reactions, alopecia, acne, increased hematocrit, and decreased testicular size (314,315). Androgen (replacement) therapy in SCI sometimes, but not always (305), is accompanied by side effects ranging from light effects such as minor skin irritations or acne (305) to more severe adverse effects, such as unfavorable changes in serum lipids and liver function tests (307). Especially the lipid changes are significant in light of the body composition changes that place individuals with SCI at greater risk for metabolic and cardiovascular disease.

To conclude, adjuvant pharmacological support with androgens in combination with resistance-type exercise (mimetics) training seems to be promising in augmenting muscle mass and strength in individuals with SCI. Further studies are warranted to elucidate the signaling pathways responsible for evoking muscle hypertrophy following exercise (mimetics) and the effect on muscle protein synthetic rates after SCI. It is very important to consider whether reported benefits of using anabolic steroids on muscle mass in the SCI population do or do not outweigh their adverse effects.

### Myostatin inhibitors

Myostatin is a member of the TGF-β family of growth factors and has been shown to reduce the size of skeletal muscle (316,317). In line with this, myostatin mRNA levels are increased in paralyzed muscle in individuals with stroke (318) as well as in those with chronic SCI (319–321). However, myostatin protein levels are not altered in people with chronic SCI (319). Long-term electrical stimulation of the soleus muscle of individuals with SCI decreased mRNA levels for myostatin (266). It would be interesting to investigate the effect of myostatin inhibitors on muscle mass in SCI, although preclinical data indicate that effective muscle growth in response to myostatin inhibition may require intact innervation (322,323). Work in the area of age-related muscle loss has applied various pharmacological interventions addressing the myostatin pathway, with many also reporting side effects such as off-target vascular effects and spontaneous bone fractures (324).

### β2 adrenoceptor agonists

Adrenoceptor agonists are substances that activate adrenoceptors. These receptors respond to adrenaline (epinephrine) and noradrenaline (norepinephrine), which are natural neurotransmitters and hormones involved in the “fight-or-flight” response. There are two main types of adrenoceptors: alpha (α) and beta (β) adrenoceptors. Agonists of these receptors cause smooth muscle relaxation, leading to effects such as bronchodilation, vasodilation in muscle and liver, and relaxation of uterine muscle. Certain β2-agonists are known to increase protein synthesis and decrease protein breakdown in skeletal muscle by activating the PI3 K/Akt/mTOR pathway and suppressing the FOXO transcriptional activation of the ubiquitin-proteosome and autophagy-lysosome pathways (325,326). In addition, β2-adrenoceptor agonists salbutamol, metaproterenol, and formoterol were shown to increase voluntary muscle force, protein turnover rates, and to induce anabolic responses in skeletal muscles in healthy men (326,327). In individuals with a C5-C7 neurological level of SCI who experienced muscle atrophy, the β2 adrenoceptor agonist metaproterenol improved muscle size and strength (328). In humans with traumatic cervical brachial plexus injury, also suffering from denervation-induced muscle atrophy, the β2-adrenoceptor agonist clenbuterol attenuated the decrease in muscle fiber CSA by approximately 60% (329). Together with some promising preclinical findings in SCI models (330–332), this supports the need for verification of these results in clinical studies involving SCI. However, it should be noted that the use of β2-adrenoceptors may cause health concerns as side effects, such as arrhythmias, elevations in blood glucose and tremor (333,334).

## Conclusions

**Figure 2:**
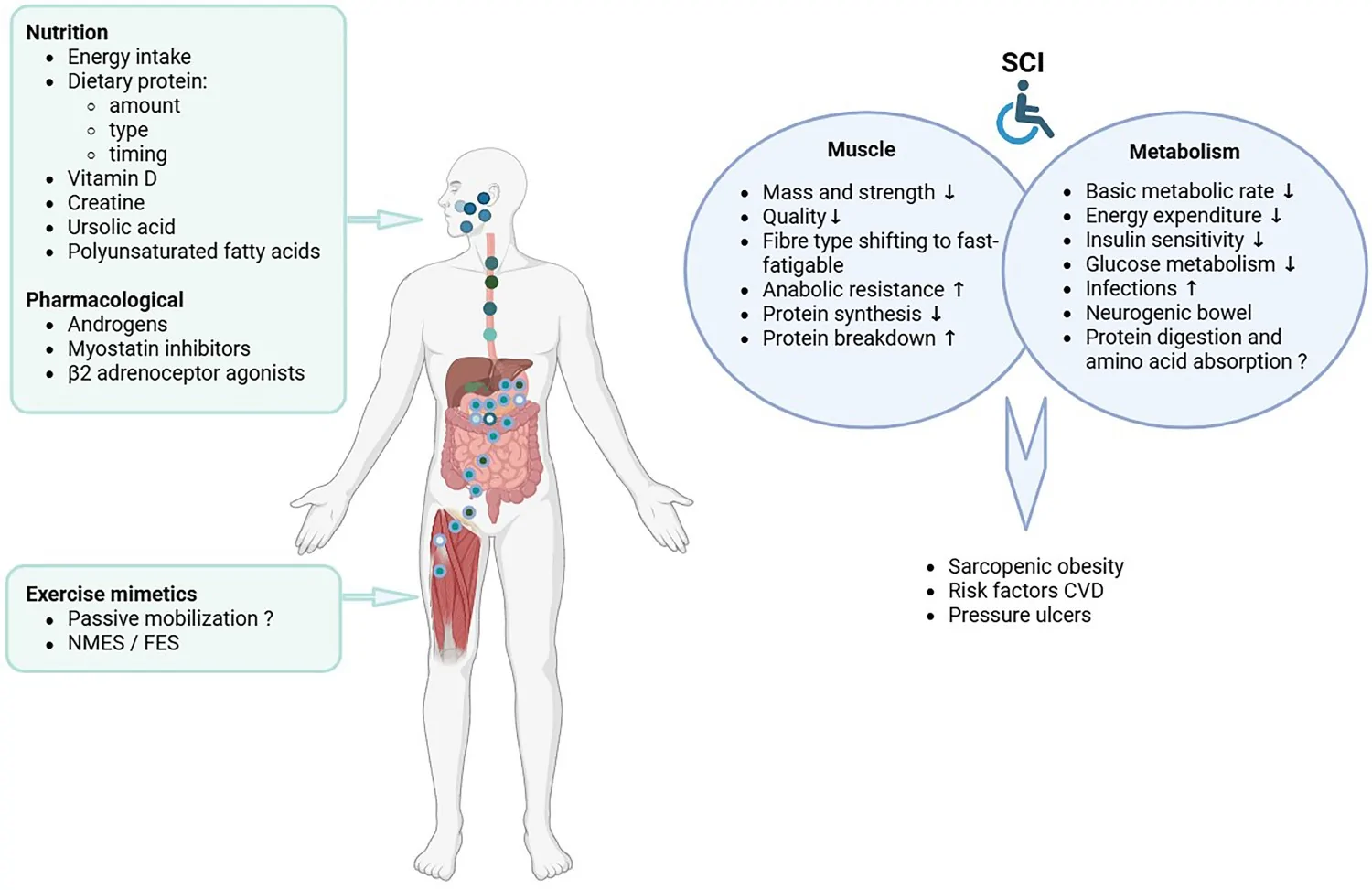
Summary of the main changes in muscle and metabolism of people with motor-complete SCI and the possible interventions (nutrition, drugs and exercise mimetics) to counteract muscle loss. *CVD Cardiovascular diseases; FES Functional electrical stimulation; NMES Neuromuscular electrostimulation.*

The findings as summarized in Fig. 2 emphasize critical considerations for managing the severe muscle mass loss in individuals with SCI. It seems highly plausible that changes in muscle protein turnover and anabolic resistance are underlying mechanisms responsible for the observed muscle atrophy. Exercise (mimetics) remain fundamental in preventing or reducing muscle atrophy. Given the fact that resistance type exercise and/or exercise mimetics reduce the anabolic resistance to protein feeding, combining NMES with protein intake is highly recommended. The amount, type, and timing of dietary protein intake need to be taken into consideration. In addition, nutritional supplements, including (a combination of) vitamin D, creatine, UA and PUFAs and/or pharmacological agents such as androgens, myostatin inhibitors, and β2-adrenoceptor agonists, may further improve muscle mass and function in SCI populations. Finally, a multidimensional approach, guided by updated clinical recommendations, could substantially enhance outcomes related to muscle mass preservation, metabolism, and functional rehabilitation in individuals with SCI. Research is warranted to assess the proposed positive effects of exercise (mimetics), nutritional strategies, and/or pharmacological interventions on muscle protein turnover and anabolic sensitivity and, as such, to mitigate muscle mass loss and prevent chronic metabolic diseases in people with complete SCI.

## Disclaimer statements

**Contributors** None.

**Funding** LJCvL has received research grants, consulting fees, speaking honoraria, or a combination of these for research on the impact of exercise and nutrition on muscle metabolism. A full overview on research funding is provided at: https://www.maastrichtuniversity.nl/l.vanloon. The other authors report there are no funding details to declare.

**Declaration of interest** None.

**Conflicts of interest** Authors have no conflict of interests to declare.
